# Double Hit of Hydroxichloroquine and Amiodarone Induced Renal Phospholipidosis in a Patient with Monoclonal Gammopathy and Sclerodermiform Syndrome: A Case Report and Review of the Literature

**DOI:** 10.3390/medsci12020025

**Published:** 2024-05-15

**Authors:** José C. De la Flor, Pablo Rodríguez-Doyágüez, Daniel Villa, Rocío Zamora, Francisco Díaz

**Affiliations:** 1 Department of Nephrology, Hospital Central Defense Gomez Ulla, 28047 Madrid, Spain; 2 Department of Nephrology, Guadalajara Center Dialysis, AVERICUM, 19003 Guadalajara, Spain; pablo.rodriguez@avericum.com; 3 Department of Nephrology, Clinica Universidad de Navarra, 28027 Madrid, Spain; devillah@unav.es; 4 Department of Nephrology, Hospital Universitario General Villalba, 28400 Madrid, Spain; rocio.zamora@hgvillalba.es; 5 Department of Anatomic Pathology, Hospital Gregorio Marañón, 28008 Madrid, Spain; fdiazc@salud.madrid.org

**Keywords:** drug-induced-phospholipidosis, zebra bodies, monoclonal gammopathy of renal significance, hydroxychloroquine, amiodarone

## Abstract

Phospholipidosis is a rare disorder which consists of an excessive intracellular accumulation of phospholipids and the appearance of zebra bodies or lamellar bodies when looking at them using electron microscopy. This disease is associated with certain genetic diseases or is secondary to drugs or toxins. Drug-induced phospholipidosis encompasses many types of pharmaceuticals, most notably chloroquine, amiodarone or ciprofloxacin. Clinically and histologically, renal involvement can be highly variable, with the diagnosis not being made until the zebra bodies are seen under an electron microscope. These findings may require genetic testing to discount Fabry disease, as its histological findings are indistinguishable. Most of the chemicals responsible are cationic amphiphilic drugs, and several mechanisms have been hypothesized for the formation of zebra bodies and their pathogenic significance. However, the relationship between drug toxicity and phospholipid accumulation, zebra bodies and organ dysfunction remains enigmatic, as do the renal consequences of drug withdrawal. We present, to our knowledge, the first case report of acute renal injury with a monoclonal gammopathy of renal significance, lesions, and sclerodermiform syndrome, with zebra bodies that were associated with the initiation of a hydroxychloroquine and amiodarone treatment, as an example of drug-induced-phospholipidosis.

## 1. Introduction

Phospholipids (PLs) are indispensable components of plasma and intracellular membranes. These portions of membranes are continuously sequestered by endocytosis and autophagy and fuse with lysosomes to be degraded and recycled. Phospholipidosis (PLD) is a disorder which consists of an excessive intracellular accumulation of PLs in the tissues [1]. In electron microscopy (EM), intracellular osmiophilic structures appearing as “lamellar bodies (LBs)” or “zebra bodies (ZBs)” or “myeloid bodies (MBs)” invariably indicate the disease [2,3,4]. The differential diagnosis of PLD includes hereditary diseases, with heterogeneous phenotypes and the progressive involvement of multiple organs such as Niemann–Pick disease, LMX1B-associated nephropathy or Fabry disease (FD) [5], while acquired or secondary PLDs are primarily iatrogenic, caused by a variety of cationic amphiphilic drugs (CADs), called Drug-induced phospholipidosis (DIP), and less frequently by intoxications such as silicone nephropathy and even contrast medium [6,7], or by other disorders such as liver cirrhosis, nephrotic syndrome, Alport syndrome or Alagille syndrome [8]. DIP has been described in relation to more than 200 chemical entities, including mainly antiarrhythmic, antidepressant, antimalarial, antipsychotic, antibiotic and antihistaminic drugs. The most frequent are chloroquine, amiodarone and ciprofloxacin. Its mechanism is not well understood; there are hypotheses related to the inhibition of lysosomal phospholipase A, and/or C, alpha-galactosidase-A (α-GLA) activity and the accumulation of indigestible drug–lipid complexes. The organs affected may exhibit histopathological changes and inflammatory reactions with deposition patterns (LBs/ZBs/MBs) in the lung, liver or kidney, where it is associated with pulmonary fibrosis, hepatic steatosis, steatohepatitis and acute or chronic kidney injury, respectively [9]. Renal PLD is an intracellular storage disease characterized by a concentric accumulation of PLs within several types of renal cells, such as mesangial, glomerular endothelial, podocytes and tubulointerstitial cells, and they can be seen by the EM of a renal biopsy (RB) [9].

FD is the most prevalent of the lysosomal storage diseases. FD is a rare X-linked disease characterized by reduced or absent α-GLA enzyme activity due to mutations in the GLA gene leading to a lysosomal accumulation of glycosphingolipids, including globotriaosylceramide-3 (GL3) and its metabolite globotriaosylsphingosine (lyso-GL3), in a variety of cells (cardiac, renal and cerebral mainly) [10]. It results in an accumulation of glycosphingolipids that give rise to these ZBs or LBs or MBs. The spectrum of the clinical manifestations of FD includes ophthalmologic signs (corneal dystrophy), cutaneous manifestations, and neurological, renal and cardiovascular alterations (complete atrioventricular-(AV) block). Chronic kidney disease (CKD) associated with proteinuria, isosthenuria, Fanconi syndrome and/or renal sinus cysts is a renal manifestation that can be seen in this metabolic disorder [10]. On occasion, DIP can mimic the histologic features of FD.

On the other hand, no case reports on the association of DIP and a monoclonal gammopathy of renal significance (MGRS) have been described in the literature. In this report, we describe an unusual case of a patient with sclerodermiform syndrome (SS) and MGRS presenting with EM-determined characteristic lesions of a renal PLD induced by the possible double effect of amiodarone and hydroxychloroquine (HCQ), mimicking FD lesions.

## 2. Case Report

A 65-year-old female with arterial hypertension (AHT) and dyslipidemia carrying a mechanical aortic valve prosthesis and dual chamber (DDD) pacemaker due to a complete AV block since August 2016. Her medication included losartan 100 mg/day, simvastatin 40 mg/day, amiodarone 200 mg/day, acenocoumarol 2 mg/day, bisoprolol 5 mg/day and hydrochlorothiazide 12.5 mg/day. Under follow-up by nephrology since June 2021 for CKD stage G3aA2, presumably secondary to hypertensive nephroangiosclerosis, with stable renal function during the previous 10 months (serum creatinine (SCr) of 1.29 mg/dL, an estimated glomerular filtration rate (eGFR) of 45 mL/min/1.73 m^2^ and urinary albumin/creatinine ratio (UACR) of 95 mg/g). In March 2022, she was diagnosed with a monoclonal gammopathy of undetermined significance (MGUS) IgG –lambda (serum IgG 1780 mg/dL, serum-free light chain ratio kappa (κ)/lambda (λ): 0.8), which is monitored by a hematologist. In September 2022, she was diagnosed with incipient SS due to the presence of pain, stiffness, induration, thickening and a distal swelling of the skin of the upper extremities and fingers (sclerodactyly), Raynaud phenomenon, arthralgia, antinuclear antibodies (ANA: 1/160) with anticentromere antibodies (1/1280) and positive capillaroscopy with unspecific alterations. There was no cardiac or pulmonary involvement and renal function remained unchanged (SCr: 1.2 mg/dL and UACR: 80 mg/g) at the time of evaluation. Its etiology, or that it is part of an overlapping syndrome with other autoimmune diseases, was not clear. Since then, she received treatment with HCQ at 400 mg/day. Two months later, she was referred to our nephrology department for an acute kidney injury (AKI) (SCr: 1.88 mg/dL), eGFR of 27.63 mL/min/1.73 m^2^ calculated by CKD-EPI, and progressive proteinuria (UACR: 550 mg/g) and a urinalysis showed a microhematuria of 20–30 red blood cells per high power field (RBC/HPF), with dysmorphic cells. Complementary tests showed the following: hemoglobin of 12 g/dL, normal haptoglobin; reticulocytes 1.26%, blood smear without schistocytes; negative rheumatoid factor (RF); and normal ADAMTS-13. No hypoalbuminemia nor hypercholesterolemia. The 24 h urine protein excretion was 1.32 g, with a spot urine-protein-to-creatinine ratio (UPCR) and UACR of 1200 and 550 mg/g, respectively. Serum protein electrophoresis (SPEP) and serum immunofixation electrophoresis (SIFE) revealed a monoclonal band IgG-lambda at 6.4% (0.39 g/dL). Urine protein electrophoresis (UPEP) and urine immunofixation electrophoresis (UIFE) revealed lambda-free light chain (λFLC) Bence Jones proteinuria. Serum λFLC and κFLC were 117.4 mg/dL and 7.8 mg/dL, respectively, with a κ/λ FLC ratio of 0.07. Hypocomplementemia C3. The rest of the immunological and viral studies are shown in Table 1. Renal ultrasound revealed a slight decrease in the size of both kidneys, moderate corticomedullary differentiation and that the renal arteries’ Doppler was normal.

Bone marrow aspiration (BMA) revealed 6% plasma cells, with negative Congo red staining. The immunophenotypic study showed that 3% of the total cellularity were plasma cells and, of these, 99.5% had an abnormal phenotype with monoclonality for the λLC restriction (CD38+, CD138+, CD45−, CD19−, CD56+, CD117+, CD27+, CD81−). Cytogenetics study (46, XX) and Fluorescence in situ hybridization (FISH) study were negative for the 13q14 region deletion, the 17p13 region deletion and the 14q32 region rearrangement. The echocardiogram was normal. A positron emission tomography–computed tomography scan (PET/CT) did not suggest lymphoproliferative syndrome, solitary plasmacytoma, or other extramedullary affectations. Bone series study showed no lytic or blastic lesions.

**Figure 1:**
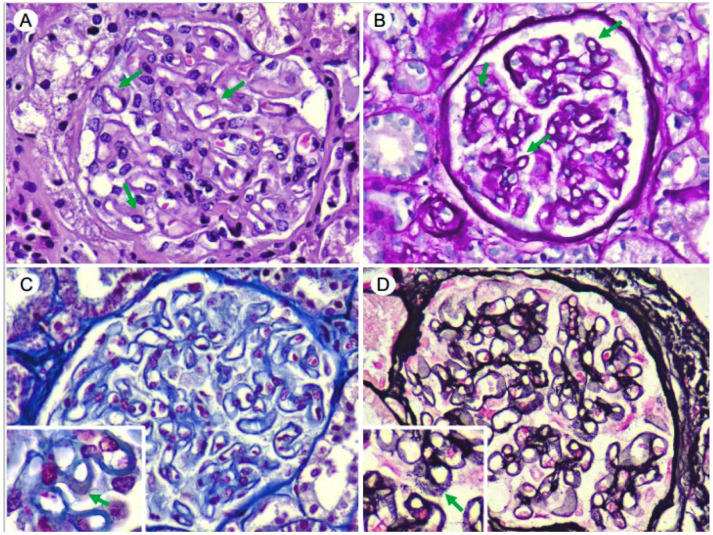
Glomerulus with thickened capillary walls, rigid aspect (arrows) (Hematoxylin–Eosin) (**A**). Periodic acid-Schiff (PAS) (**B**), with granular fuchsinophilic deposit (arrow) (Masson’s trichrome) (**C**). The methenamine–silver technique shows basement membranes with a moth-eaten appearance with spicular projections towards the sub-epithelial aspect (arrow) (**D**). (×40).

**Figure 2:**
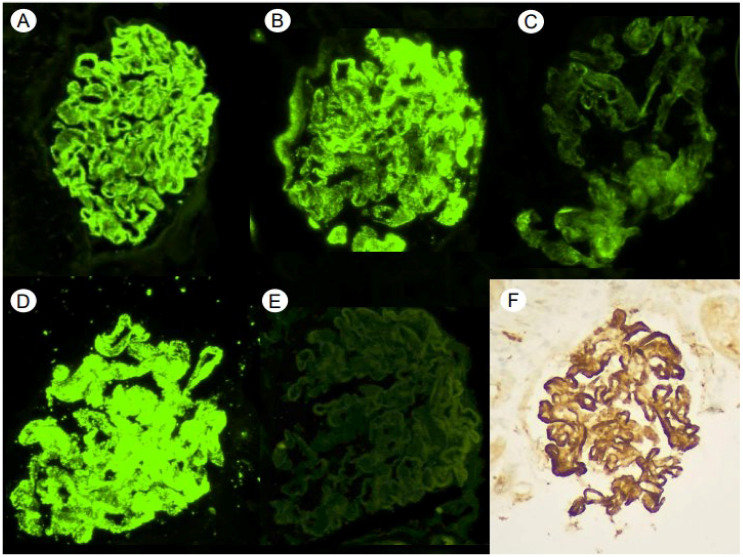
Direct immunofluorescence techniques show the overall deposition, with a predominantly sub-epithelial granular pattern, in the glomerular capillary loops and, to a lesser extent, the mesangial, positive for IgG (3+) (**A**), C3 (3+) (**B**) and C1q (1+) (**C**), with a restriction for Lambda light chains (3+) (**D**). Negative Kappa light chains (**E**). Immunohistochemistry technique for the detection of C4d shows intense positivity with the same pattern (**F**) (×40).

As renal involvement of the known gammopathy was assumed, an RB was performed. Light microscopy (LM) showed 15 glomeruli, 8 of which were globally sclerosed; the remaining showed thick glomerular basement membranes with subepithelial “spikes”. Additionally, a fine cytoplasmatic vacuolization of podocytes was observed. There was no endocapillary or extracapillary proliferation. There was minimal (10%) interstitial fibrosis with proportional tubular atrophy (Figure 1A–D). A cytoplasmic vacuolation of renal tubule epithelium was also observed. Arteries and arterioles were unremarkable. Congo red and thioflavin staining were negative. The frozen tissue immunofluorescence (IF-F) study showed positive capillary wall granular and subepithelial staining for IgG (3+), C3 (3+), C1q (+) and λLC (3+) with restriction. IF-F was negative for IgA, IgM, C4 and fibrinogen. IgG subtyping was not performed (Figure 2A–E). The immunohistochemical staining study was negative for Anti-Phospholipase A2 Receptor Antibody (Anti-PLA2R) and IgG4, while it was positive for C4d (Figure 2F). Based on the clinical, analytical and histological findings of LM and IF without obtaining, at that time, data from EM, the diagnosis was MGRS of the atypical membranous nephropathy (MN) type, with IgG-λ deposition. In view of the above findings, a treatment was started with cycles of bortezomib, cyclophosphamide and dexamethasone chemotherapy.

**Figure 3:**
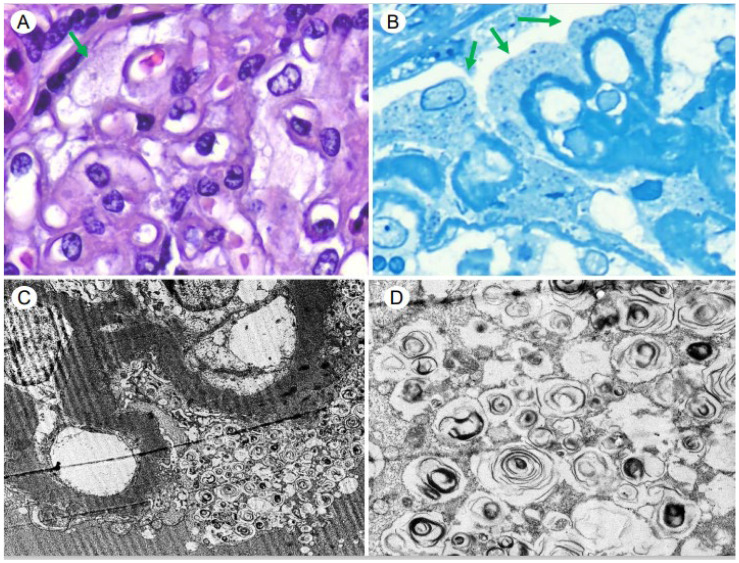
Glomerulus with hypertrophic podocytes with vacuolated cytoplasms (arrows) (Hematoxylin–Eosin, ×60) (**A**) (Toluidine Blue, ×60) (**B**) and with multifocal presence of intracytoplasmic inclusions that are markedly electron-dense (Electron Microscopy, ×2000) (**C**) and arranged to form concentric sheets with a “zebroid” or “myelinaceous” appearance (Electron Microscopy, ×8000) (**D**).

**Figure 4:**
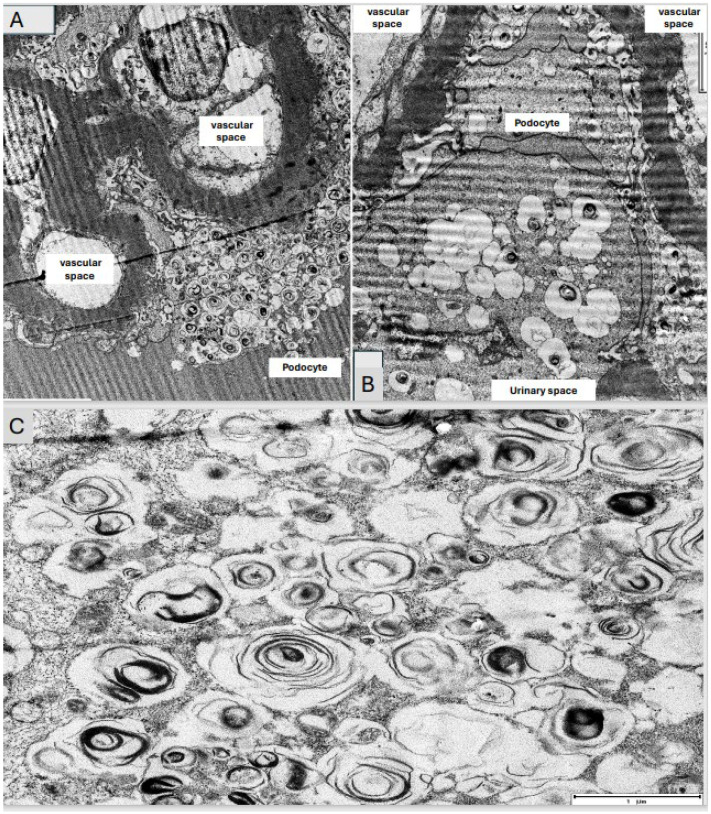
Electron microscopy showing enlarged podocyte with numerous large lipid inclusions (zebra bodies); the capillary wall is mildly thickened (original magnification ×2000) (**A**,**B**). Numerous concentric multilaminated inclusions (myeloid bodies) in the cytoplasm of the podocytes (**C**).

In December 2022, the results of an EM study revealed electron-dense ZBs/LBs/MBs forming concentrically in the cytoplasm of the podocytes and epithelial cells of the Bowman’s capsule, with an ultrastructural diagnosis of intrapodocyte inclusions. As described, these bodies are typically found in PLD (Figure 3A–D and Figure 4A–C). Therefore, the patient and her daughters underwent a α-GLA functional and genetic enzymatic study (DNA extraction from a dried blood spot, PCR amplification and sequencing of all coding exons and flanking intronic regions) for FD. The serum α-GLA and lyso-GL3 levels reported were 1.6 μmol/mL/h (normal controls > 2.8 μmol/L/h) and 0.4 ng/mL (normal controls 0.0–3.5 ng/mL), respectively. Although the patient had a complete AV block and CKD, findings that lead to a suspicion of FD, the genetic study did not detect a pathogenic mutation, so it was concluded that the ZBs were compatible with DIP and the diagnosis of FD was discarded. A few days later, the patient was admitted to the hospital for acute respiratory distress syndrome (ARDS) and worsening kidney function (SCr: 2.5 mg/dL). After discarding infectious and immunologic causes, a clinical and radiologic suspicion of diffuse acute interstitial pneumonia (AIP) due to amiodarone/HCQ vs. bortezomib was reached. However, due to the poor clinical condition of the patient, a surgical lung biopsy could not be performed. If, under EM, MBs/ZBs/LBs were present in alveolar macrographs, this would have supported the diagnosis of AIP due to a PLD induced by amiodarone and HCQ. Despite the withdrawal of these drugs, there was no improvement in kidney function (SCr: 2.38 mg/dL) and, two weeks later, the patient died of cardiorespiratory causes.

## 3. Discussion

We reported the case of a patient with a recent diagnosis of incipient SS and an MGRS of a type atypical of NM, with -IgG-λ deposition. An ultrastructural examination showed the presence of renal PLD lesions that mimic the morphological findings observed in FD. Histologically, the LM and IF of our patient’s RB showed features of glomerular involvement with an NM pattern and granular and subepithelial staining for IgG (3+), C3 (3+), C1q (+) and LCλ (3+) in the IF, which is uncommon in renal PLD, where null or slight staining is expected [11]. The 2019 expert consensus document of the International Kidney and Monoclonal Gammopathy Research Group [12] mentions that MGRS-associated renal lesions are initially separated by the presence or absence of MIg deposits in their RB samples. Furthermore, according to the ultrastructural characteristics of the deposits, they are subclassified into organized and non-organized [13]. The latter are seen in patients with monoclonal immunoglobulin deposition disease (MIDD), proliferative glomerulonephritis with monoclonal immunoglobulin deposits (PGNMID) and the miscellaneous category, including monotypic MN or monotypic anti-glomerular basement membrane disease [12,13]. NM, as a histologic lesion of MGRS, has been described in only a few case reports associated with PGNMID lesions (5% of cases) [14] and MIDD [15,16]. Cases with exclusively membranous features have been referred to as atypical MN with LC restrictions. Best et al. [17] reported 28 cases of MN with an LC isotype restriction. The authors conclude that the absence of Anti-PLA2R, a positive staining for a single IgG subclass and the presence of focal proliferation are histopathologic features that should prompt a clinical workup to exclude the presence of an underlying lymphoproliferative disorder. In our patient we found all these data, i.e., the presence of a monoclonal IgG lambda band in serum and urine, negative anti-PLA2R staining, a history of MGUS-IgGλ and the detection of a pathologic plasma IgGλ clone in BMA, associated with the histologic features already mentioned, led to the conclusion of a diagnosis of MGRS of an atypical NM type with IgG-λ deposition.

However, the most relevant findings from the renal histology of our patient were those found in the ultrastructural study. EM showed the presence of electron-dense lamellar deposits, MBs/ZBs/LBs, within the cytoplasm of the podocytes, Bowman’s capsule epithelial cells and tubular epithelium, changes classically attributed to FD. The differential diagnosis of deposits under EM includes other renal lipidoses of hereditary origin, iatrogenic intoxications and drug toxicity, such as DIP with α-GLA inhibition [8]. As described in the literature, DIP can cause ultrastructural histological changes that mimic the typical findings of FD [18]. The MBs or ZBs or LBs found in biopsies are the same in both diseases and it is virtually impossible to distinguish a DIP from FD by their morphological characteristics. Choung HYG et al. [11] retrospectively reviewed 4000 native RBs obtained at the University of Rochester Medical Center (URMC)’s Pathology Laboratory (URMC) from 2010 to 2021 to identify the presence of MBs/ZBs/LBs under EM. They identified 32 cases with MBs, separating the cases into two groups: those who had a diagnosis of FD (FDG) (6 patients) and another group without FD (non-FDG) (26 patients). They also randomly selected 22 cases with neither FD nor MBs/ZBs/LBs as a control group (non-MBG) to compare their medication information with that of the non-FDG group. The mean age in the FDG and non-FDG groups was 48 years (range 38–59) and 56 years (range 11–85), respectively. All were female in the FDG and there was a female predominance in the non-FDG (female/male ratio: 15/11). Of the non-FGD group, 15 patients had a history of hypertension and 8 had diabetes mellitus. Proteinuria was the most frequent RB indication in the FDG and non-FDG groups (50% and 83%, respectively), followed by AKI (42% and 50%). There were no significant differences between the FDG and non-FDG groups with respect to proteinuria (slightly higher in non-FDG), sCr and eGFR. They compared the medication list between the non-FDG and non-MBG groups and they found no significant differences except for hydralazine and sertraline, which were identified more frequently in the non-FDG group (*p* = 0.04). In addition, 83% of patients in the non-FDG and 86% of those in the non-MDG group were taking at least one drug reported to be a cause of DIP. From an ultrastructural point of view, non-FDG patients showed predominantly focal MB/ZB/LB lesions (podocytes), while FDG cases showed more extensive MB/ZB/LB lesions not only in podocytes, but also in parietal, tubular, endothelial cells and myocytes (*p* = 0.03). These data were accordant with the EM findings of our patient and with the series of cases reported by de Menezes et al. [18]. Therefore, it is suspected that MBs/ZBs/LBs are usually more numerous in FD than in DIP, with the inclusions being small, round and granular. Although it is very difficult to distinguish the type of involvement of renal cells caused by MBs/ZBs/LBs, it is usually described that, in the case of DIP, it is limited to the podocytes and, in that of FD, other cells are usually affected, not just the podocytes; endothelial cells and the smooth muscle fibers of the vascular walls may also be involved [18]. Another relevant fact from Choung HYG’s study [11] was that they found that more than half of the FDG cases had another superimposed kidney disease, including LC-kappa deposition disease (1 patient), thin basement membrane nephropathy (1 patient), and lithium-related changes (1 patient), while the RB’s diagnoses of the non-FDG patients were hypertensive arterionephroesclerosis (53%), followed by acute tubular necrosis (27%), diabetic glomerulosclerosis (19%), ANCA-associated pauci-immune glomerulonephritis (15%), lupus nephritis (12%) and acute interstitial nephritis (8%). They conclude that MBs/ZBs/LBs are not only present in FD but also other environments including DIP (mainly caused by aminoglycosides, hydroxychloroquine and amiodarone), toxins and other hereditary diseases. Although secondary causes of MBs/ZBs/LBs usually show their less extensive involvement compared to FD, these features overlap [11]. On the other hand, our case does not describe the intralysosomal inclusions which are frequently seen in FD. The cytoplasmic vacuolation of podocytes is also an EM finding in renal PLD induced by chloroquine toxicity. Despite this, its pathologic features are not specific and require clinical and molecular correlation, especially to exclude FD-like features [11,18]. Therefore, the clinical presentation, the patient’s family history, extra-renal symptoms and biochemical features help to differentiate these two entities. The diagnosis of FD in males can be made by measuring the enzyme activity in their peripheral blood or leukocytes alongside the characteristic findings in EM. However, given the challenges in diagnosing female carriers of FD, the finding of MBs/ZBs/LBs, although not specific to this disease, may be the only clue that leads to its more detailed study and timely evaluation. In these cases, it is necessary for the diagnosis include a study of the genetic mutation, since the enzyme analysis conducted in the blood or leukocytes may be normal [19]. In addition, a subsequent genetic analysis discounted this hereditary disorder, directing the diagnostic compass towards DIP, with a particular incrimination of amiodarone and HCQ, which are cationic amphiphilic drugs (CADs). Therefore, to our knowledge, this is the first case report where RB describes DIP and MGRS lesions.

Regarding the clinical manifestations, our patient showed an increase in proteinuria with the disproportion between her UACR and UPCR associated with AKI, and the results of the immunological study (viral serology, autoimmunity study, paraproteinemia, etc.) initially did not allow us to discard the presence of an overlapping systemic autoimmune disease such as SLE on SS versus MGRS. However, the patient did not meet the criteria according to the 2019 EULAR/ACR classification for SLE [20], and the IF on her RB reasonably discarded lupus nephritis (no full house staining, no strong staining for C1q). As for other drug-related nephrotoxicities apart from DIP, her usual medication, as well as HCQ, have not been associated with secondary NM, and the tubulo-interstitial involvement was too scarce and non-specific to suggest drug-related tubulo-interstitial nephritis. Our view is that the fact that the patient had SS is not related to her renal involvement or to the development of renal PLD. First, our patient was diagnosed with SS with positivity for anti-centromere antibodies and ANA, which is less related to renal involvement; there was no involvement of other organs such as the lungs or heart; and there was no suspicion of scleroderma renal crisis (SRC) in the absence of hypertension and vascular involvement in the RB [21]. We could not to find any association between DIP, SS and GMSR in the literature, so we think that their relationship has a casual rather than causal effect. However, paraneoplastic scleroderma-like disorders, which have been described in the context of certain plasma cell dyscrasias, could not be discarded as a possible association with MGRS [22]. Therefore, after the diagnosis of MGRS, a chemotherapy treatment directed at the pathological clone was initiated, without clinical or analytical improvement. Despite this, our patient presented with other more than notable respiratory complications. The renal and extra-renal complications of autoimmune or onco-hematologic diseases can be triggered by poor control of the disease or be related to its treatment. In our case report, the ultrastructural study of the patient’s EM showed findings of renal PLD, a disease characterized by abnormal lipid storage. Some cases of AKI by renal PLD induced by CADs have been described [19,23], but, in general, there is no clear association between renal PLD and negative consequences for renal function [24]. It is important to mention that the patient’s GFR remained stable while she was taking amiodarone, which she had since 2016, and only worsened 4 weeks after initiating the treatment with HCQ. The functional consequence of renal PLD and its association with toxicity are still poorly understood [1,25,26]. CADs (i.e., amiodarone, chloroquine and aminoglycosides) are the main causes of PLD [24]. To date, 13 cases of chloroquine or HCQ-induced renal PLD have been reported in the literature, most of them in the context of autoimmune diseases such as systemic lupus erythematosus and Sjogren’s syndrome [27]. All cases occurred in women with an age range between 14 and 70 years. A total of 76% of the cases received HCQ at an average dose of 400 mg/day, while the rest of the cases received chloroquine [27]. The data agree with the clinical history of our patient. However, reported cases of amiodarone-induced renal PLD are rare; Pintavorn et al. [23] reported one case of a 71-year-old African American woman with CKD (SCr 1.6 mg/dL) who began treatment with amiodarone for a cardiac arrhythmia diagnosed 4 months earlier during a hospital admission for interstitial pneumonia of unknown etiology. The analysis highlighted a significant AKI (SCr 3.5 mg/dL), so an RB was performed, in which the presence of ZBs was evident in the ultrastructural study. Given the suspicion of FD, an enzymatic and genetic study of α-GLA was carried out, finding no mutations associated with this disease. The patient did not have symptoms nor a family history of FD, except for the presence of cornea verticillata. During questioning, it was discovered that the patient had been mistakenly taking a high dose of amiodarone. The final diagnosis was amiodarone-associated renal and corneal PLD mimicking FD. After the discontinuation of amiodarone after 3 months, her SCr improved to 2.4 mg/dL. A study in male rats treated with amiodarone for weeks observed the accumulation of PLs in lung, kidney and skeletal muscle, and, in the long term, in other organs such as the heart, observing a significant correlation between increased phospholipids and decreased thyroid function due to amiodarone [28]. Amiodarone has been shown to be a phospholipase A1 and A2 inhibitor, by inhibiting the electrostatic charge interactions between hydrolase and anionic phospholipids [23,28]. Oikawa et al. [29] reported the case of an 85-year-old male with liver cirrhosis under treatment with therapeutic doses of amiodarone and whose liver biopsy showed numerous lysosomes with dense lamellar inclusions. The discontinuation of amiodarone led to a slight improvement in his serum aminotransferase levels. However, to date, there are no cases of renal PLD induced by the combination of amiodarone and HCQ, except for our case report. There are some things that are not clear in our patient; one of them is the exposure time for the development of renal PLD induced by HCQ. Most case reports refer to several years of exposure, while in the case of amiodarone, the case described by Pintavorn et al. [23] mentions short times and high doses, data that do not agree with our patient, who was treated with amiodarone for a long period (since 2016) and, on the contrary, with HCQ for a short time (only 4 weeks before the onset of kidney disease intervention). It is difficult to be sure that the synergism of both CADs (HCQ and amiodarone) is the cause of the formation of MBs/ZBs/LBs or, on the contrary, whether it is only in relation to amiodarone. We hypothesize that both CADs may have reached high concentrations within the lysosome, inhibiting phospholipase at concentrations significantly higher than those associated with their therapeutic activity, promoting the formation of inclusions in the kidney tissue.

Several mechanisms have been hypothesized in DIP [1,9,26]. CADs bind directly to phospholipids to give rise to non-digestible drug–lipid complexes, which accumulate in the lysosomes in the form of these LBs/ZBs/MBs. Within the intralysosomal vesicles (where sphingolipid degradation occurs), there is an inhibition of the action of hydrolases that, under normal conditions, catabolize different lipids, such as phospholipase A1 (PLA1) and A2 (PLA2), phospholipase C (PLC), sphingomyelinase and other related enzymes. There are several theories about such enzymatic inhibitions. The first one would be explained by a direct binding to and inhibition of these lysosomal proteins, although this is not clear [26]. The second would be explained by the fact that CADs, due to their structure with a hydrophobic, often aromatic ring and a slightly hydrophilic head group that can be protonated at its weak base, can enter the membranes of the intralysosomal system. Once they interact with intralysosomal vesicles (ILVs), which are negatively charged, they become protonated and, as positively charged amphiphiles, they are trapped within the acidic compartment and accumulate. The compensation for the negative surface charge of the ILVs decreases the efficiency of the catabolic proteins like sphingolipid and glycosphingolipid degrading hydrolases; phospholipases A1, A2 and C; acid sphingomyelinase; and lipases, which require a negatively charged substrate to operate (Mingeot-leclercq theory) [1,9,28]; or PLA2 [23] and triggers their release from the ILVs to the lysosomal membrane. There, these catabolic proteins would be easily degraded by lysosomal proteases, further reducing the catabolism of most lipids (phospholipids, sphingolipids and neutral glycerolipids) and greatly decreasing the export of cholesterol from the lysosomes. The decreased catabolic capacity of the lysosomes and the accumulation of lipids and hydrophobic material converts the affected lysosomes into dysfunctional storage granules, LBs [1,26]. The other theory focuses on the deregulation of genes related to lipid synthesis, PLA activity or enzymatic transport [1]. There is no clear evidence of other possible mechanisms in the pathogenesis of DIP, such as an increased biosynthesis of cholesterol or phospholipids, or a direct inhibition of lysosomal phospholipases by all these CADs. On the other hand, alveolar macrophages (AMs) can collect ZBs/MBs/LBs, giving rise to foamy macrophages with the activation of the immune response. One theory sees DIP as an adaptive response to drug exposure rather than a toxic consequence as part of the detoxification mechanism, since drug entrapment by lysosomes, the inhibition of lysosomal enzymatic activity and the subsequent expulsion of the lamellar body by exocytosis would prevent excessive oxidation within the cell, protecting it from toxicity [1,9]. This is contradicted by the study performed by Reasor et al. [30], in which they administered amiodarone to rats, inducing PLD in their AMs. Using this model, various pulmonary defense functions were evaluated in the host. The pulmonary clearance of Listeria monocytogenes by macrophages with PLD was not affected. Furthermore, using an ex vivo culture system of AMs, it was seen that the presence of PLD had no effect on the phagocytosis of dead yeast cells, and the spontaneous release of interleukin-6 (IL-6) or tumor necrosis factor alpha (TNFα) was improved compared to cells without PLD. Thus, in the context of the functions studied, the induction of pulmonary PLD did not appear to alter the pulmonary host defense processes in rats. In our case, we could not prove that the patient’s AIP was caused by this DIP.

Recently, in vitro screening methods for DIP based on gene expression analyses have been suggested, identifying numerous genes as possible markers for DIP and contrasting them with different concentrations of drugs that we know cause DIP to identify their potential to trigger it. Significant reproducibility has been observed in the different studies that have been conducted [31]. In our report, no genetic study was performed, so we cannot contrast our case with the emerging evidence.

## 4. Conclusions

In conclusion, our report highlights the imperative for clinicians to maintain a high index of suspicion for DIP in patients presenting with renal abnormalities and to carefully review medication history as a routine component of the diagnostic workup, mainly in situations where common genetic conditions are mimicked. This is particularly relevant for drugs like amiodarone, HCQ and aminoglycosides, which have a well-documented history of DIP. It also serves as a critical reminder of the interplay between clinical judgment and diagnostic investigations, asserting the necessity for a comprehensive and meticulous approach to patient care. The interrelationships between DIP and other systemic diseases such as SS and dysproteinemic kidney disease (MGRS) are yet to be fully understood and, as this is a single case report, our findings may not be generalizable to other patients with similar conditions and more research is needed. Lastly, this case advocates for the importance of a multidisciplinary approach to complex cases, integrating clinical, pathological and ultrastructural analyses to achieve an accurate diagnosis and thereby inform appropriate management strategies.

## Figures and Tables

**Table 1:** Laboratory findings on admission.

		Reference Range/Unit
WBC	5980	U/L
Hemoglobin (Hb)	12	12–16 g/dL
Platelet count (Plt)	170	10^3^/uL
Reticulocytes count	1.26	2–4%
Erythrocyte count	3.43	4.2–5.8 × 10^6^/uL
Lactate dehydrogenase (LDH)	385	135–214 IU/L
Coombs Test	Negative	NA
Total Bilirubin	0.69	0.1–1 mg/dL
Total protein	6.5	6.4–8.7 g/dL
Serum Albumin (Alb)	3.84	3–5.5 g/dL
GOT	32	5–32 IU/L
GPT	24	5–33 IU/L
Triglycerides	85	30–150 mg/dL
Total cholesterol	175	110–200 mg/dL
Urea	73	17–60 mg/dL
Creatinine	1.88	0.6–1.2 mg/dL
Na	140	135–145 mmol/L
K	4.5	3.5–5.5 mmol/L
Cl	103	95–110 mmol/L
P	4.1	2.5–4.5 mg/dL
Serum calcium total	8.8	8–10.4 mg/dL
CRP	0.70	0.1–0.5 mg/dL
Hbs-Ag	Negative	NA
HCV-Ab	Negative	NA
HIV	Negative	NA
Cryoglobulins	Negative	NA
C3	86	90–180 mg/dL
C4	18.1	10–40 mg/dL
RF	Negative	<15 IU/ml
ANA	1/160	NA
Antids-DNA	< 1	0–20 UI/ml
Anti-Sm, Anti-RNP, Anti-Ro/SSA, Anti-La/SSB, Anti-Scl-70, Anti-jo-1, Anti-chromatin antibodies, anti-Rib-*P*	Negative	NA
Antiphospholipid antibodies (APL): aCL IgG antibody Lupus anticoagulant B2GP1 IgG antibody	50 Negative Negative	<15 GPL U/mL 20–39 GPL U/mL ≤20 SGU U/mL
Anti-centromere antibody	1/1280	NA
ANCA and cryoglobulin	Negative	NA
Anti-GBM	Negative	<1 AI
Anti-PLA2R Ab (ELISA)	Negative	NA
Beta 2 microglobulin	0.63	<0–20 mg/dL
IgG	1980	800–1600 mg/dL
IgA	36.3	70–400 mg/dL
IgM	46.6	90–180 mg/dL
UPCR	1200	<20 mg/g
UACR	550	<30 mg/g
Urine red blood cells	20–30	/HPF
24 h urine total protein excretion	1.32	<0.15 g/24-h
SPEP M-protein concentration	6.4%	NA
SIFE	IgG-lambda	NA
UPEP/UIFE	IgG-lambda	NA
κFLC	7.8	4.90–13.70 mg/L
λFLC	117.4	7.60–19.50 mg/L
κ/λ FLC	0.07	0.27–1.67

AI: activity index, AU: arbitrary unit, NA: not applicable, WBC: white blood cell, GOT: Glutamate-Oxaloacetate Transaminase, GPT: glutamate pyruvate transaminase, Na: sodium serum, K: potassium serum, Cl: chloride serum, P: phosphorus serum, CRP: C—reactive protein, C3: Complement 3, C4: Complement 4, RF: rheumatoid factor, ANA: Antinuclear antibody, Antids-DNA: anti-double-stranded DNA antibody, Anti-Sm: Anti-smith, Anti-RNP: anti-U1 ribonucleoprotein, Anti-Ro/SSA: antibody Ro- Sjögren’s syndrome A, Anti-La/SSB: antibody *La*- Sjögren’s syndrome B, Anti-Scl-70: Scl-70 antibody, Anti-jo-1: jo1 antibody, anti-Rib-*P*: anti-*ribosomal* *P* (anti-Rib-P) antibodies, APL: antiphospholipid antibody, ANCA: anti-neutrophil cytoplasmic autoantibody, Anti-GBM: anti-glomerular basement membrane, Anti-PLA2R Ab: Anti-Phospholipase A2 Receptor Antibody, Ig: Immunoglobulin, UPCR: spot urine-protein-to-creatinine ratio, UACR: spot urine-albumin-to-creatinine ratio, ELISA: Enzyme-Linked Immunosorbent Assay, SPEP: Serum protein electrophoresis, SIFE: Serum immunofixation electrophoresis, UPEP/UIFE: urine protein electrophoresis/urine immunofixation electrophoresis, FLC: free light chain, κ: kappa, λ: lambda, HPF: high-power field.

## Data Availability

No new data were created or analyzed in this study. The data used to support the findings of this study are available from the corresponding author on request (contact J.C.D.l.F., josedelaflor81@yahoo.com, jflomer@mde.es). We confirm that all the figures and tables are the original work of this manuscript’s authors. All have been performed by the authors of this manuscript, have not been adapted from other authors, and do not have an online link.
